# A chromEM-staining protocol optimized for cardiac tissue

**DOI:** 10.3389/fcell.2023.1123114

**Published:** 2023-07-05

**Authors:** Elettra Musolino, Christina Pagiatakis, Federica Pierin, Daniele Sabatino, Giovanna Finzi, Rosalba Gornati, Giovanni Bernardini, Roberto Papait

**Affiliations:** ^1^ Department of Biotechnology and Life Sciences, University of Insubria, Varese, Italy; ^2^ Department of Cardiovascular Medicine, Humanitas Research Hospital–IRCCS, Rozzano (MI), Italy; ^3^ Department of Pathology, ASST Sette Laghi, Varese, Italy

**Keywords:** chromEM, chromatin structure, heterochromatin, cardiac tissue, aging

## Abstract

Three-dimensional (3D) chromatin organization has a key role in defining the transcription program of cells during development. Its alteration is the cause of gene expression changes responsible for several diseases. Thus, we need new tools to study this aspect of gene expression regulation. To this end, ChromEM was recently developed: this is an electron-microscopy staining technique that selectively marks nuclear DNA without altering its structure and, thus, allows better visualization of 3D chromatin conformation. However, despite increasingly frequent application of this staining technique on cells, it has not yet been applied to visualize chromatin ultrastructure in tissues. Here, we provide a protocol to carry out ChromEM on myocardial tissue harvested from the left ventricles of C57BL/6J mice and use this in combination with transmission electron microscopy (TEM) to measure some morphological parameters of peripheral heterochromatin in cardiomyocytes. This protocol could also be used, in combination with electron tomography, to study 3D chromatin organization in cardiomyocytes in different aspects of heart pathobiology (e.g., heart development, cardiac aging, and heart failure) as well as help to set-up ChromEM in other tissues.

## 1 Introduction

Cell-type identities depend on specific gene expression programs that are established during development (Agelopoulos et al., 2021). Crucial in defining these programs is three-dimensional (3D) chromatin organization, a phenomenon that regulates the accessibility of proteins involved in transcription (e.g., transcription factors and other proteins of the transcription machinery) to DNA (Ko et al., 2008; Chapski et al., 2019; Trzaskoma et al., 2020). Moreover, dysregulated 3D chromatin organization can trigger gene expression changes that cause disease, such as cancer, neuronal disorders, and cardiovascular diseases (Ito et al., 2014; Zhou et al., 2019; Papait et al., 2020; Hu et al., 2021; Kim et al., 2022). Therefore, it is not surprising that different approaches have been developed to study 3D chromatin organization in physiological and pathological conditions (Cremer et al., 2015; Hausmann et al., 2020). Among these, there is considerable interest in electron-microscopy-based techniques that allow not only the visualization of DNA–DNA contacts between genomic regions that can be distant from each other along the primary structure of the DNA strand, but also reveal information on the physical structure of the chromatin and how it is spatially organized in the nucleus (Trzaskoma et al., 2020; Zhou et al., 2021; Yusuf et al., 2022). Indeed, thanks to the short wavelength of the accelerated electrons of the microscope beam (which is much shorter than that of the photons of the visible spectrum), the resolution limit of a conventional transmission electron microscope can go down to about 0.2 nm. As far biological samples are concerned, however, the ability to appreciate ultrastructural details is mainly limited by the properties of the sample rather than by the resolution of the instrument. Indeed, samples have to be prepared not only to maintain their structural stability with efficient fixation, but also to obtain enough contrast (that is, a difference in intensity between adjacent points) to render biological features distinguishable. This is usually achieved with heavy metals such as lead, uranium, and osmium that generate an amplitude contrast due to their mass. To this aim, therefore, chromatin must be adequately and specifically contrasted. Unfortunately, lead citrate and uranyl acetate–the conventional chromatin stains–do not bind specifically to DNA, and the chromatin-staining technique based on the Feulgen-like osmium amine reaction–which has the advantage of selectively labeling DNA, allowing better definition of chromatin organization–can damage chromatin structure due to its acidity (Ou et al., 2017).

**FIGURE 1:**
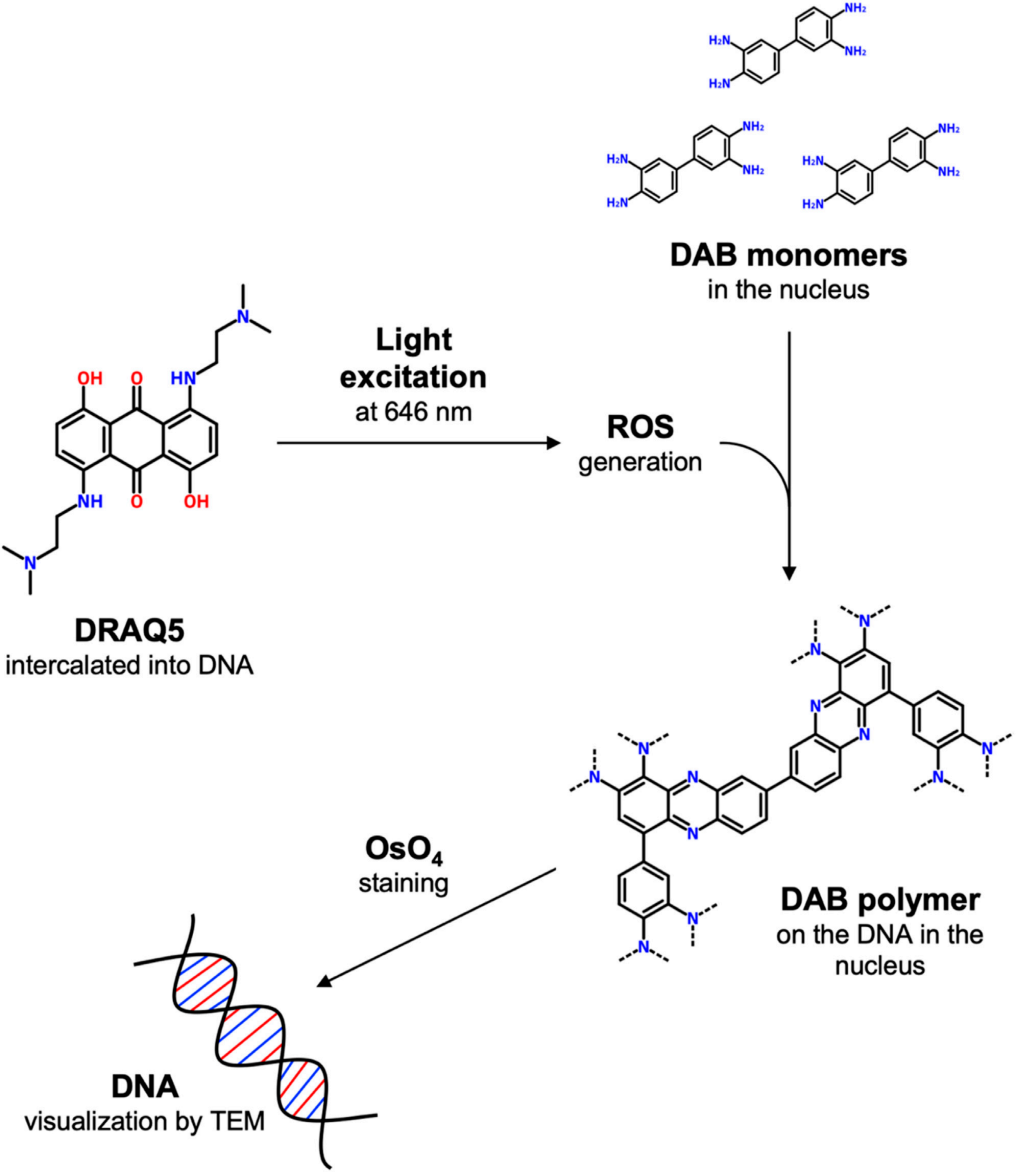
Schematic representation of ChromEM staining. Briefly, when excited at 646 nm, DRAQ5 intercalated into DNA catalyzes the polymerization of DAB monomers through local generation of reactive oxygen species. Polymerized DAB appears as a dark precipitate on the DNA that can be enhanced with osmium tetroxide (OsO_4_) staining.

To overcome these limits, ChromEM was recently developed (Ou et al., 2017). This staining technique selectively marks nuclear DNA without altering its structure, leading to a better visualization of 3D chromatin conformation. This technique is based on the labeling of DNA with DRAQ5 (1, 5-bis{[2-(di-methylamino)ethyl]amino}-4, 8-dihydroxyanthracene-9, 10-dione), a cell-permeable far-red fluorescent dye that intercalates double-stranded DNA. When excited at 646 nm, DRAQ5 catalyzes the polymerization of DAB (3,3′-diaminobenzidine) through local generation of reactive oxygen species. Polymerized DAB appears as a dark precipitate that can be enhanced with osmium tetroxide (OsO_4_) staining (Figure 1).

ChromEM staining combined with electron microscopy tomography was used to show how the chromatin in human osteosarcoma cells and in a small-airway epithelial cell line is not organized in a hierarchical pattern, but is found as 5–24 nm thick fibers. Higher order structures with diameters of 40–50 nm and 100–130 nm have been observed to date only *in vitro* (Ou et al., 2017). Moreover, ChromEM staining in association with electron microscopy or electron tomography has been used to visualize the organization of DNA within the nucleus of other eukaryotic cell lines (Tonnemacher et al., 2020; Li et al., 2021b; 2021a). For example, in human epidermal progenitor cells, mechanical stretch was shown to significantly reduce heterochromatin associated with the nuclear envelope, a phenomenon confirmed by a decreased level of H3K9me3, a histone modification associated with heterochromatin (Nava et al., 2020). It was also successfully used to visualize the compacted circular chromosomal DNA of *Synechococcus elongatus* PCC 7942 (Ghosh et al., 2021). However, despite increasingly frequent application of this staining technique on cells, ChromEM has not yet been applied to visualize chromatin ultrastructure in tissues.

Cell lines are a powerful tool in cell biology, but they have two strong limitations: firstly, they do not capture the complexity and heterogeneity of tissues; secondly, these cells are somewhat altered with respect to those from which they were derived, a consequence of *in-vitro* culturing. Thus, the study of chromatin in tissues is fundamental if we have to fully understand the role of 3D chromatin architecture in regulating gene expression in physiological and pathological conditions. Here, we provide a protocol to carry out ChromEM on left-ventricle myocardium harvested from 6-month-old C57BL/6J mice. We think that this protocol could help in the study of 3D chromatin organization in cardiomyocytes during heart development, cardiac aging, and heart failure in mouse as well in human biopsies. In addition, our results could help to set up ChromEM staining in other tissues.

## 2 Materials and equipment

### 2.1 Reagents

• Potassium chloride (P3911, Sigma-Aldrich)
• Glutaraldehyde solution–Grade II, 25% in H_2_O (G6257, Sigma-Aldrich)
• Paraformaldehyde (387507, Carlo Erba Reagents)
• Sodium phosphate monobasic–ReagentPlus^®^, ≥99.0% (S0751, Sigma-Aldrich)
• Sodium phosphate dibasic–ReagentPlus^®^, ≥99.0% (S0876, Sigma-Aldrich)
• Sodium Cacodylate Buffer—0.4 M, pH 7.2 (11655, Electron Microscopy Sciences)
• Agarose (A5093, Sigma-Aldrich)
• Glycine–ReagentPlus^®^, ≥99.0% (G7126, Sigma-Aldrich)
• Saponin (47036, Sigma-Aldrich)
• DRAQ5™ (ab108410, Abcam)
• Hydrochloric acid (HCl)—ACS reagent, 37% (320331, Sigma-Aldrich)
• 3,3′-Diaminobenzidine (DAB) tetrahydrochloride hydrate (D5637, Sigma-Aldrich)
• Osmium tetroxide (OsO_4_)—2% aqueous solution (19152, Electron Microscopy Sciences)
• Ethanol absolute–ACS Grade, Scharlab (ET00232500, Fisher Scientific)
• Propylene oxide–ReagentPlus^®^, ≥99.0% (110205, Sigma-Aldrich)
• Embed 812 (14900, Electron Microscopy Sciences)
• D. D.S.A. (13700, Electron Microscopy Sciences)
• NMA (19000, Electron Microscopy Sciences)
• DMP-30 (13600, Electron Microscopy Sciences)
• Sodium citrate dihydrate (W302600, Sigma-Aldrich)
• Lead (II) nitrate–ACS reagent, ≥99.0% (228621, Sigma-Aldrich)
• Sodium hydroxide–reagent grade, ≥98%, pellets (S5881, Sigma-Aldrich)
• Uranyl acetate · 2H_2_O (77870, Serva)

### 2.2 Solutions

• KCl: 50 mM potassium chloride in physiological H_2_O
• Sodium phosphate monobasic stock solution: 0.2 M NaH_2_PO_4_ in dH_2_O
• Sodium phosphate dibasic stock solution: 0.2 M Na_2_HPO_4_ in dH_2_O
• 0.2 M sodium-phosphate buffer pH 7.2: 23% sodium phosphate monobasic stock solution, 77% sodium phosphate dibasic stock solution
• Karnovsky’s fixative: 2.5% glutaraldehyde, 2% paraformaldehyde, 0.1 M sodium-phosphate buffer pH 7.2 in dH_2_O
• Blocking buffer: 10 mM glycine in 0.1 M sodium cacodylate buffer
• Saponin: 0.1% saponin in 0.1 M sodium cacodylate buffer
• DRAQ5: 10 µM DRAQ5 in 0.1% saponin
• DAB: 2.5 mM DAB, 10 mM HCl in 0.1 M sodium cacodylate buffer
• Osmium tetroxide: 1% OsO_4_ in 0.1 M sodium cacodylate buffer
• Resin: 45% Embed 812, 30% D. D.S.A., 23% NMA, 2% DMP-30
• Lead citrate: 0.1 M sodium citrate, 0.1 M lead nitrate, 16% sodium hydroxide in dH_2_O
• Uranyl acetate: supersaturated solution of uranyl acetate in dH_2_O

### 2.3 Instruments

• Vibratome (Sorvall TC-2 tissue sectioner)
• Full-spectrum lamp (150 LEDs Full Spectrum Grow Light 100W)
• Molds–Embedding Mold-Dykstra, 15 × 7 × 4 mm (70907, Electron Microscopy Sciences)
• Ultramicrotome (Pabisch TOP-Ultra 170)
• Diamond knife–DiATOME ultra 45° (27-US, Electron Microscopy Sciences)
• Grids–Gilder Grids, 400 lines/inch square mesh (EMS400-Au, Electron Microscopy Sciences)
• Transmission electron microscope (Philips Morgagni 268D)

## 3 Setting up of the protocol

To set up a procedure for ChromEM staining of myocardial tissue, we used left ventricles from 6-month-old C57BL/6J mice. The prerequisite for successful ChromEM staining is to slice tissue into sections thin enough so that, after full saponin permeabilization, there is complete penetration into the sample of all the reagents necessary for the staining reaction. Indeed, sample dimensions can influence the timing of DRAQ5 staining (indispensable to localize DAB at the nuclear level) and of DAB photo-oxidation (essential to obtain good DNA contrast). For mouse cardiac muscle, we found that samples of 2 × 3 mm in size and with a thickness of 100 µm were small enough to allow adequate reagent permeation while remaining large enough for optimal handling. However, appropriate dimensions should be defined for each type of tissue under investigation and kept constant during experiments to obtain comparable results.

**FIGURE 2:**
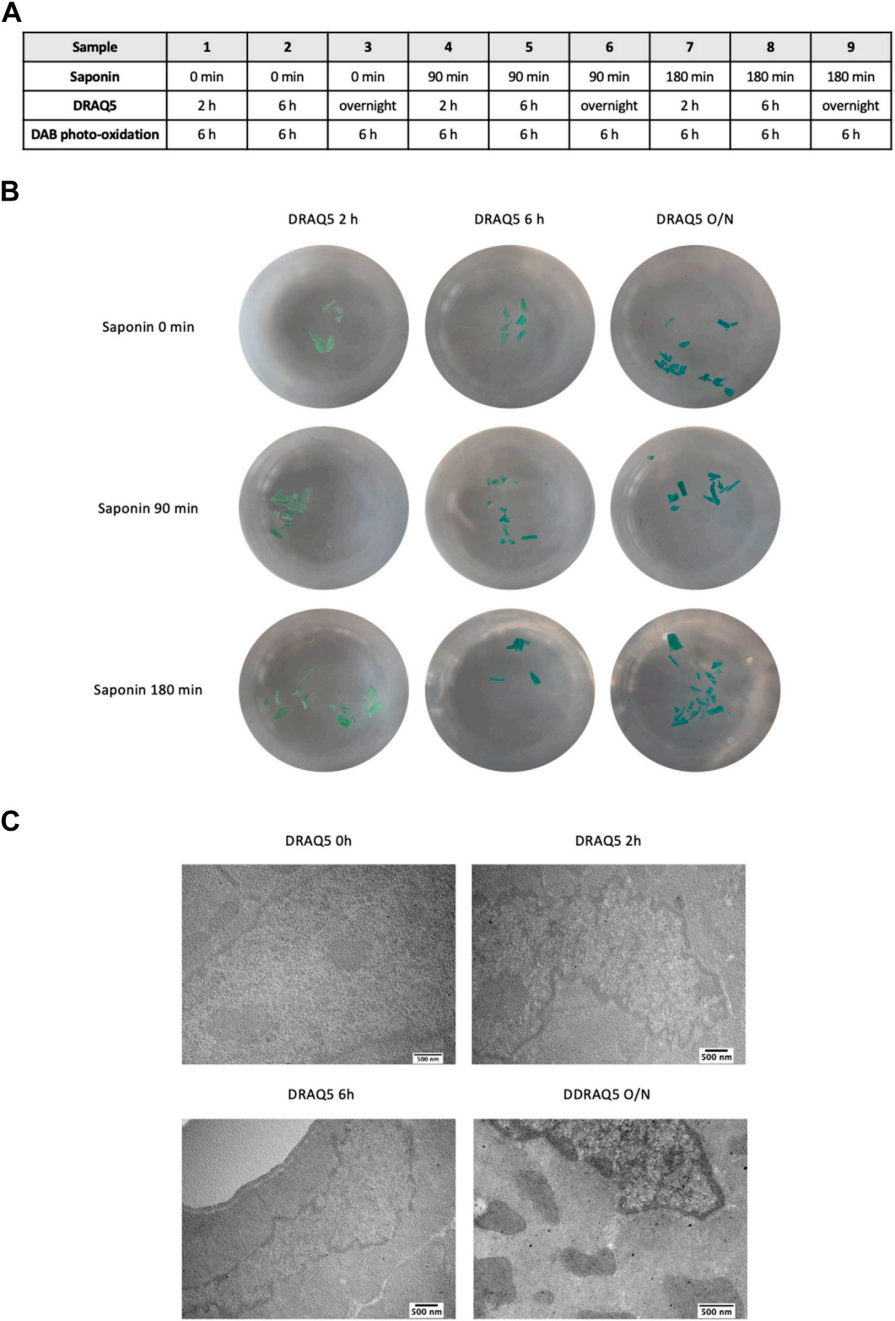
**(A)** Experimental scheme used to determine the most efficient incubation times for saponin and DRAQ5 in cardiac tissue. **(B)** Images of cardiac tissue sections treated for 2 h, 6 h, and overnight with DRAQ5 with and without a prior permeabilization step for 90 or 180 min with saponin. Photo-oxidation of DAB was fixed at 6 h for all nine tests **(C)** TEM images of cardiac tissue after 0 h, 2 h, 6 h, and overnight with DRAQ5 and a prior permeabilization step for 90 min with saponin.

Next, we determined the best incubation times for saponin, DRAQ5, and DAB photo-oxidation. To define the most efficient incubation times for saponin (set at 0, 90, or 180 min) and DRAQ5 (set at 2h, 6h, or overnight), we fixed photo-oxidation of DAB at 6 h (Figure 2A). Increased exposure of the samples to DRAQ5 resulted in better staining, but pretreatment with 0.1% saponin appeared to neither enhance dye penetration nor alter cell and chromatin morphology within our tissue (Figures 2B,C). In light of these results, the final protocol includes a 90 min pretreatment step with saponin.

**FIGURE 3:**
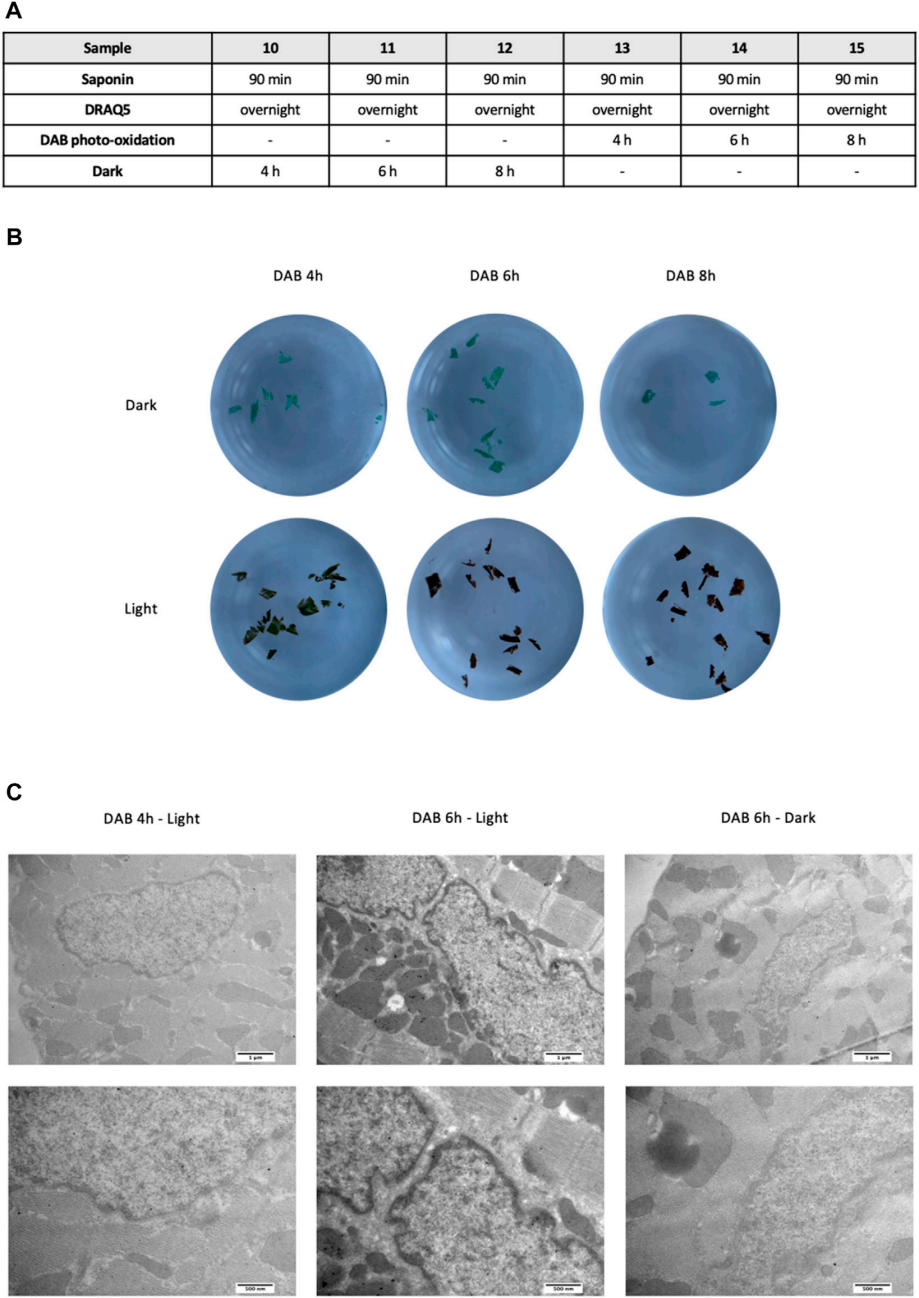
**(A)** Experimental scheme used to define the most efficient length of time for DAB photo-oxidation in cardiac tissue. **(B)** Macroscopic differences between 4, 6, and 8 h of photo-oxidation, with saponin permeabilization fixed at 90 min before incubation with DRAQ5 overnight for all samples. Samples incubated with DAB but kept in the dark for the indicated times were used as controls **(C)** TEM images of cardiac tissue after 4h and 6 h of DAB in the light (left) and after 6 h of DAB in the dark (right) at two different magnifications.

Then, to determine the best length of time for photo-oxidation, sections were incubated with DAB and exposed to light for 4, 6, or 8 h, keeping constant saponin incubation and DRAQ5 staining times; as negative controls, we used sections incubated with DAB but kept in the dark (Figure 3A). Since photo-oxidation causes a change in color from blue to brown, we first evaluated the efficiency of the reaction by analyzing the color of the sections: 4 h of light exposure was not enough for complete photo-oxidation of DAB, as a blue undertone persisted; 6 h of photo-oxidation was necessary for the development of a homogeneous brown color throughout the section (Figure 3B). This was confirmed by observation of the samples under TEM. Indeed, chromatin had a more visible and defined contrast in sections subjected to 6 or 8 h of photo-oxidation than in sections treated for 4 h and in the negative controls (Figure 3C).

The longer incubation time with DRAQ5 and of photo-oxidation compared to Ou’s protocol is due to the greater thickness of tissue with respect to single cells and to the presence in cardiomyocytes of myofibrils which form a network so dense as to make it more difficult for the reagents to penetrate, especially in the innermost layers of the 100-μm sections. Tissues that have a less compact structure may require shorter incubation times to obtain good DNA contrast.

**FIGURE 4:**
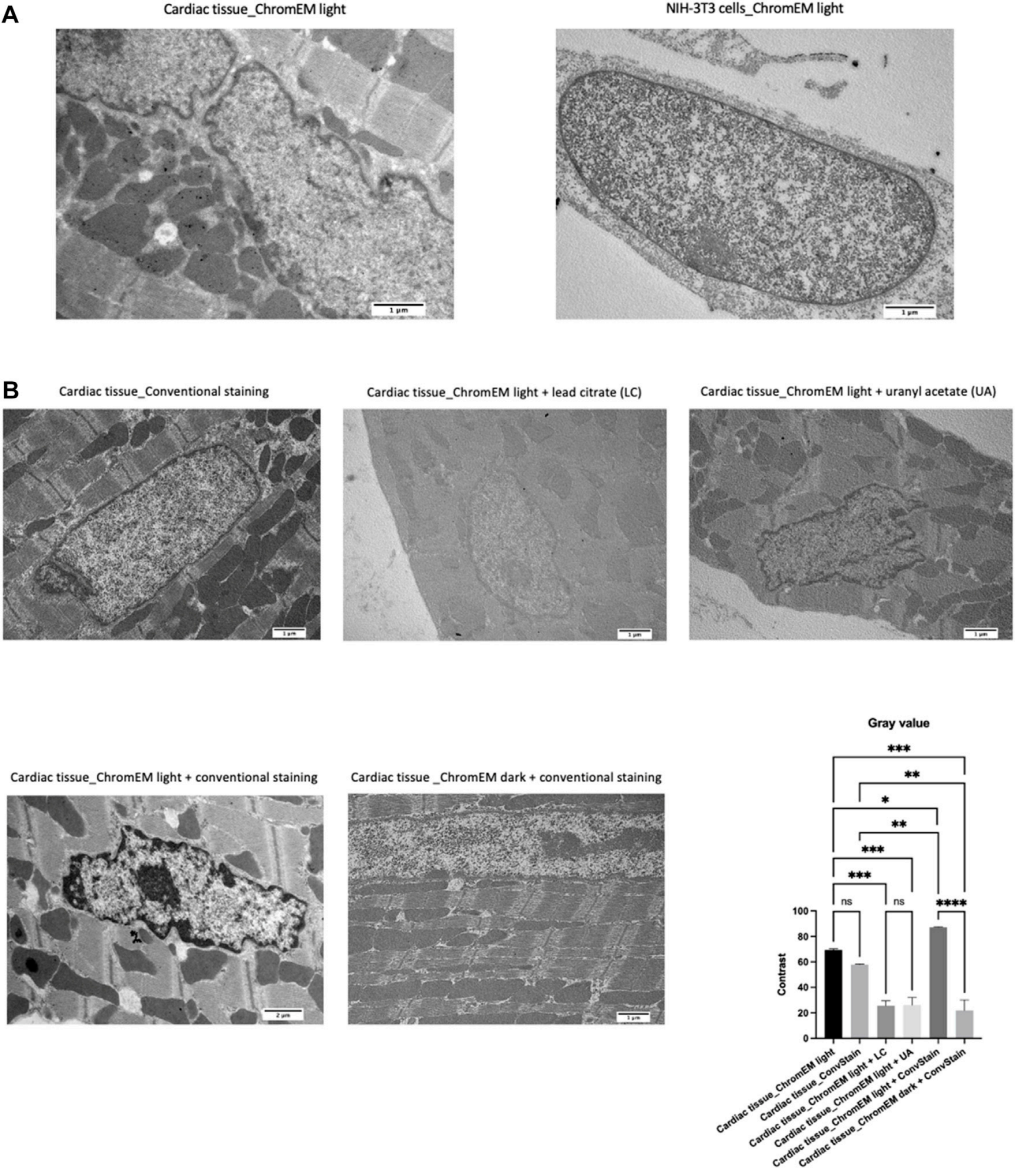
**(A)** TEM images of cardiac tissue (top left) and NIH-3T3 cells after ChromEM staining (top right). **(B)** Cardiac tissue after conventional staining (lead citrate plus uranyl acetate) and after ChromEM staining (saponin 90 min, DRAQ5 O/N, and DAB 6 h) in the presence of light with lead citrate only (top center), uranyl acetate only (top right), lead citrate and uranyl acetate (bottom left), and after ChromEM staining in the dark with subsequent lead citrate plus uranyl acetate staining (bottom center). The bar graph shows the contrast value that was calculated by measuring the difference between the mean gray value of the sarcomeres with the mean gray value of the peripheral heterochromatin of the same cells, selecting for each staining type 12 areas (6 to evaluate the gray value in peripheral heterochromatin regions, and 6 to evaluate the gray value in sarcomeric regions).

Despite the significant differences observed between controls and ChromEM-stained tissue samples, DNA contrast quality was still slightly lower than that obtained with the protocol of Ou and others (Ou et al., 2017) on the murine fibroblast cell line NIH-3T3 (Figure 4A). Therefore, in an attempt to improve chromatin visualization in our myocardial sections, we assessed the outcome of adding a staining step with conventional staining (6 min lead citrate, then 25 min uranyl acetate, then 6 min of lead citrate again), which stains molecules involved in the organization of chromatin fibers, such as proteins and RNAs (Supplementary Figure S1). The contrast was expressed as the difference between the mean gray values of sarcomeres and peripheral heterochromatin of the same cells (Figure 4B). This double staining produced better visualization of chromatin, allowing for more accurate measurement of some morphological parameters, such as the areas of the nucleus occupied by euchromatin and heterochromatin, the thickness of the chromatin blocks and the area of heterochromatin associated with the inner membrane of the nuclear envelope.

However, since uranyl ions bind phosphate groups in DNA and RNA, there is the possibility, until the due checks are made, that ChromEM associated with conventional staining produces an RNA/DNA signal overlap. Therefore, one limit of double staining is that it may hinder the study of DNA organization at different chromatin compaction levels. Thus, the choice of combining chromEM with a staining step utilizing lead citrate and uranyl acetate should depend on the type of chromatin parameters under investigation.

## 4 Finalized protocol

### 4.1 Tissue preparation

• After anesthetizing the mouse, perform surgical excision of the heart by cutting the aorta just below the thymus.
• Cannulate the aorta, fix the cannula to a syringe and wash the heart one time with physiological H_2_O and one time with 50 mM KCl.
• Immediately perfuse the heart with 2 mL of Karnovsky’s fixative.
• Proceed with removal of the atria and isolation of the left ventricle.
• Fix the ventricle by immersing it in Karnovsky’s fixative for an additional 48 h.
• Rinse the left ventricle twice in 0.1 M sodium cacodylate buffer at 4°C.

**PAUSE STEP:** either proceed with the protocol or store the left ventricle at 4 °C in 0.1 M sodium cacodylate buffer.

### 4.2 Vibratome sectioning

• Cut 2 × 3 mm portions of left ventricle using a scalpel.
• Embed the left ventricle portions in 5% agarose.
• Cut 100-µm-thick sections of left ventricle on a vibratome, placing the sample’s major axis perpendicular to the vibratome blade.

NOTE depending on sample dimensions and section thickness, the subsequent incubation times for DRAQ5 and DAB could change. Indeed, the smaller the sample, the shorter the times required for DRAQ5 to penetrate it and stain DNA, and for DAB to reach the nucleus to be photo-oxidized.

• Separate the tissue sections from the agarose with the help of tweezers, being careful not to damage the samples.
• Place the sections in the wells of a 24-well-plate and rinse with 0.1 M sodium cacodylate buffer (5 washes of 2 min each on a shaker).

**PAUSE STEP:** either proceed with the protocol or store the tissue sections at 4 °C in 0.1 M sodium cacodylate buffer.

### 4.3 ChromEM staining

NOTE unless otherwise specified, all the steps below are performed at room temperature with the use of solutions at 4°C.

• Incubate the tissue sections in blocking buffer for 15 min while shaking.
• Rinse the sections in 0.1 M sodium cacodylate buffer (5 washes of 2 min each on a shaker).
• Permeabilize the tissue with 0.1% saponin for 90 min while shaking.
• Stain the DNA with 10 µM DRAQ5 overnight at 37 °C while shaking.
• Remove excess dye by rinsing sections in blocking buffer (3 washes of 10 min each on a shaker).
• Dip the sections in 2.5 mM DAB.
• Photo-oxidize the DAB in the sample for 6 h using a full-spectrum lamp.
• After photo-oxidation, rinse the sections in 0.1 M sodium cacodylate buffer (5 washes of 2 min each on a shaker).
• Transfer each sample to a 1.5 mL tube.

**PAUSE STEP:** store the tissue at 4 °C in 0.1 M sodium cacodylate buffer until the next day.

### 4.4 Sample preparation for transmission electron microscopy

• Post-fix the sections in 1% OsO_4_ for 1 h.
• Rinse the sections in 0.1 M sodium cacodylate buffer (3 washes of 10 min each)
• Dehydrate the sections in increasing concentrations of cold ethanol: 30% EtOH for 15 min, 50% EtOH for 15 min, 75% EtOH for 15 min, 95% EtOH for 30 min, and lastly 100% EtOH for 90 min (3 washes of 30 min each).
• Incubate the sections in propylene oxide (2 washes of 10 min each).
• Incubate the sections in a 1:1 mixture of propylene oxide and resin for 90 min.
• Incubate the sections into pure resin overnight.
• Transfer the sections to embedding molds and add pure resin.
• Allow resin to polymerize at 60 °C for 48 h in an oven.
• After resin polymerization, cut 70-nm ultra-thin sections with an ultramicrotome by means of a diamond knife.

NOTE it is possible that ultrathin sections at different depths have slightly different contrast, so it would be better to always cut the tissues at the same depth from the vibratome-section surface.

• Place the ultra-thin sections on grids.

**PAUSE STEP:** store the grids at RT and protected from light until lead citrate and uranyl acetate staining.

### 4.5 Lead citrate and uranyl acetate staining

• Stain the ultra-thin sections by placing the grids on droplets of filtered lead citrate for 6 min.

NOTE create droplets of lead citrate in Petri dishes and add some NaOH pellets laterally in order to prevent precipitation of lead citrate through exposure to CO_2_.

• Rinse the ultra-thin sections with filtered dH_2_O (5 washes).
• Stain the ultra-thin sections by placing the grids on droplets of filtered uranyl acetate for 25 min in the dark.
• Rinse the ultra-thin sections with filtered dH2O (5 washes).
• Stain the ultra-thin sections by placing the grids on droplets of filtered lead citrate for 6 min.
• Rinse the ultra-thin sections with filtered dH_2_O (5 washes).
• Dry the grids with bibulous paper.

**PAUSE STEP:** store the grids at RT and protected from light until image acquisition.

### 4.6 Image acquisition and analysis

• Acquire the images of ventricular ultra-thin sections using a transmission electron microscope at 80 kV.
• Analyze the images of cardiomyocyte nuclei with Image J or similar software. In our case, the “freehand selections” tool was used to measure the area and the perimeter of the nucleus and the minor axis of peripheral heterochromatin (Supplementary Figure S2A); the “freehand lines” tool was used to measure the length of the internal nuclear membrane in contact with peripheral heterochromatin (Supplementary Figure S2B); and the “wand tool” command was used to select and measure the area of peripheral heterochromatin and heterochromatin dispersed in the nuclei (Supplementary Figure S2C,D). The specifications used for this last command were manually adjusted for each measurement, using the “tolerance” parameter.
• Perform statistical analysis of measurements made using GraphPad Prism or similar software. In our case, after verifying the normal distribution of the data with Kolmogorov-Smirnov test, significance was determined using one-way ANOVA test.

## 5 Results and discussion

**FIGURE 5:**
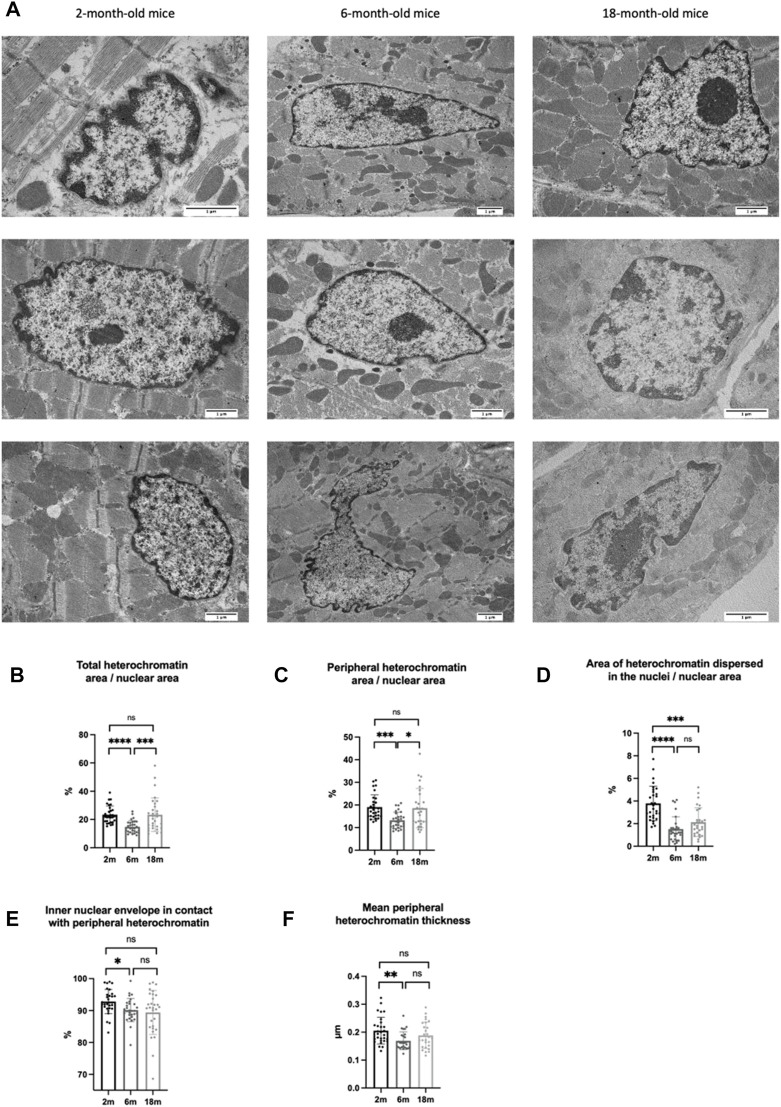
**(A)** Cardiomyocyte nuclei from mice at different ages (2-, 6-, and 18-month-old mice) at TEM after ChromEM with subsequent lead citrate plus uranyl acetate staining. **(B)** Percentage that the total area of heterochromatin (peripheral heterochromatin plus heterochromatin domains dispersed in the nucleus) occupies within the respective nucleus area. **(C)** Percentage that the total area of peripheral heterochromatin occupies within the respective nucleus area. **(D)** Percentage that the total area of heterochromatin domains dispersed in the nucleus occupies within the respective nucleus area **(E)** Percentage of the inner nuclear membrane in contact with peripheral heterochromatin. **(F)** Mean thickness of peripheral heterochromatin. Each dot corresponds to a nucleus.

We used this protocol to investigate whether chromatin organization changes in cardiomyocytes during aging. To this end, we quantified heterochromatin area in the nucleus of cardiac cells from the left ventricle of three C57BL/6J mice for each of the three stages of life analyzed: young (2-month-old mice), mature adult (6-month-old mice), aging-onset (18-month-old mice) (Hagan, 2017) (Figure 5A).

Heterochromatic regions are characterized by highly condensed chromatin and are transcriptionally inactive (Tamaru, 2010; Oh et al., 2022). To investigate if heterochromatin organization changes during aging, we quantified the area occupied by peripheral heterochromatin and by heterochromatin dispersed in the nuclei. We found that the transition from the young stage to the mature adult one is associated with decreases in peripheral heterochromatin and heterochromatin domains dispersed in the nuclei. However, only peripheral heterochromatin area increased at the onset of aging (Figures 5B–D). To verify whether this increase was also associated with a different peripheral heterochromatin organization in the nuclei, we measured the percentage of the inner nuclear membrane in contact with peripheral heterochromatin as well as the mean thickness of the heterochromatin layer, finding statistically significant decreases in both parameters during the transition from the young to the adult stage. Although the percentage of inner nuclear membrane in contact with peripheral heterochromatin continued to decrease at the onset of aging, the average thickness of the peripheral heterochromatin layer slowly began to increase (Figures 5E,F). Thus, the increased peripheral heterochromatin area in cardiomyocytes at the onset of aging could be due to deepening of the heterochromatin associated with the nuclear envelope towards the center of the nucleus. These results suggest that the reorganization of peripheral heterochromatin could underpin the gene expression changes causing impairment of cardiac function, a main cause of heart failure in the elderly. To verify this hypothesis, future studies will be carried out to answer the following questions: A) is peripheral heterochromatin redistribution required for cardiac aging? B) which cellular pathways are triggered by peripheral heterochromatin reorganization? and C) is peripheral heterochromatin redistribution associated with changes in chromatin folding?

Of note, since this protocol can be carried out even on a tiny amount of tissue, which is easily obtainable from biopsies, we think that it could help to understand the nuclear organization of chromatin in human tissues, an aspect often difficult to study with 3D genome mapping methods such as Hi-C, HiChIP, and ChIAPET, which require a large number of cells (Xu and Liu, 2019; Trzaskoma et al., 2020; Kumar et al., 2021). This is of utmost importance for many reasons. For example, chemical substances, including nanoparticles used in various industrial and medical fields, could have toxic effects on one or more epigenetic mechanisms (Paunovic et al., 2021). We are convinced that ChromEM staining could became a powerful tool for the monitoring of epigenetic toxicity through its effect on chromatin conformation (Musolino et al., 2022).

## Data Availability

The raw data supporting the conclusion of this article will be made available by the authors, without undue reservation.

## Supplementary material

SUPPLEMENTARY FIGURE S1
**(A)** Representative TEM images of cardiac tissue after conventional staining (lead citrate plus uranyl acetate), **(B)** after ChromEM staining (saponin 90 min, DRAQ5 O/N, and DAB 6h) in the dark with subsequent lead citrate plus uranyl acetate staining, and after ChromEM staining in the presence of light **(C)** with subsequent lead citrate and uranyl acetate, **(D)** lead citrate only, and **(E)** uranyl acetate only.

SUPPLEMENTARY FIGURE S2
**(A)** The area and perimeter of nuclei and the minor axis of the peripheral heterochromatin were measured in cardiomyocytes using the "freehand selections" tool of ImageJ. **(B)** The length of the internal nuclear membrane in contact with peripheral heterochromatin was measured using the "freehand lines" tool of ImageJ. The area of peripheral heterochromatin **(C)** and heterochromatin dispersed in the nuclei **(D)** were measured using the “wand tool” command of ImageJ.
