# Supplementary material for: A chromEM-staining protocol optimized for cardiac tissue
Source: Front Cell Dev Biol. 2023 Jul 5;11:1123114. doi: 10.3389/fcell.2023.1123114 (PMC10354428; doi:10.3389/fcell.2023.1123114)
Supplement: Supplementary file 1 [file Image1.pdf]

A ChromEM-staining protocol optimized for cardiac tissue

Elettra Musolino[1], Christina Pagiatakis[2], Federica Pierin[1], Daniele Sabatino [3], Giovanna Finzi[3], Rosalba Gornati[1], Giovanni Bernardini[1], Roberto Papait[1,2*]

[1]Department of Biotechnology and Life Sciences, University of Insubria, Varese, Italy

[2] Department of Cardiovascular Medicine, Humanitas Research Hospital - IRCCS, Rozzano (MI), Italy

[3]Department of Pathology, ASST Sette Laghi, Varese, Italy

*** Correspondence:**
Roberto Papait: roberto.papait@uninsubria.it

Supplementary figures

Figure S1. (A) Representative TEM images of cardiac tissue after conventional staining (lead citrate plus uranyl acetate), (B) after ChromEM staining (saponin 90 min, DRAQ5 O/N, and DAB 6h) in the dark with subsequent lead citrate plus uranyl acetate staining, and after ChromEM staining in the presence of light (C) with subsequent lead citrate and uranyl acetate, (D) lead citrate only, and (E) uranyl acetate only.

Figure S2. (A) The area and perimeter of nuclei and the minor axis of the peripheral heterochromatin were measured in cardiomyocytes using the "freehand selections" tool of ImageJ. (B) The length of the internal nuclear membrane in contact with peripheral heterochromatin was measured using the "freehand lines" tool of ImageJ. The area of peripheral heterochromatin (C) and heterochromatin dispersed in the nuclei (D) were measured using the “wand tool” command of ImageJ.

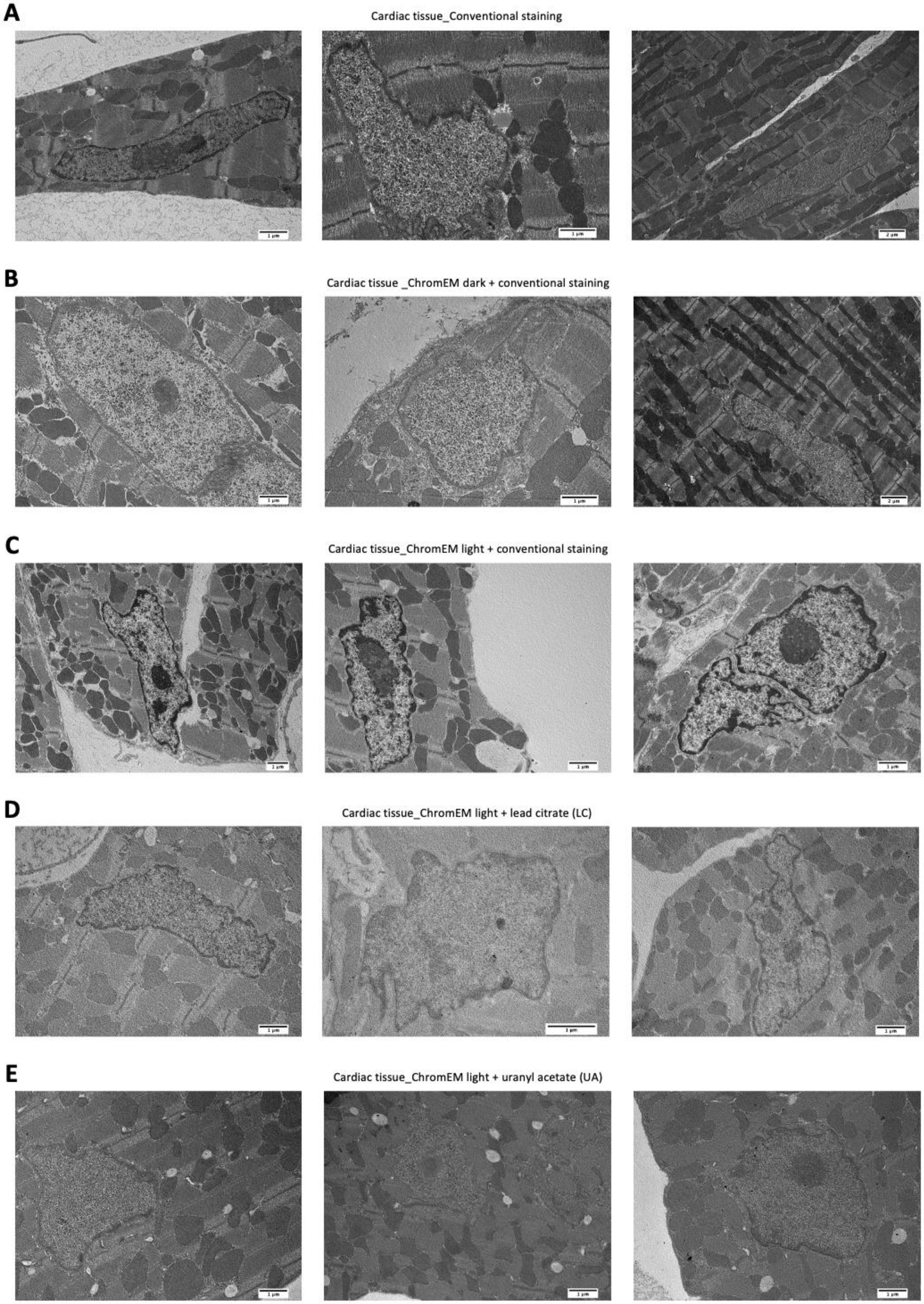
A
Cardiac tissue_Conventional staining
B
Cardiac tissue _ChromEM dark + conventional staining
C
Cardiac tissue_ChromEM light + conventional staining
D
Cardiac tissue_ChromEM light + lead citrate (LC)
E
Cardiac tissue_ChromEM light + uranyl acetate (UA)

Figure S1

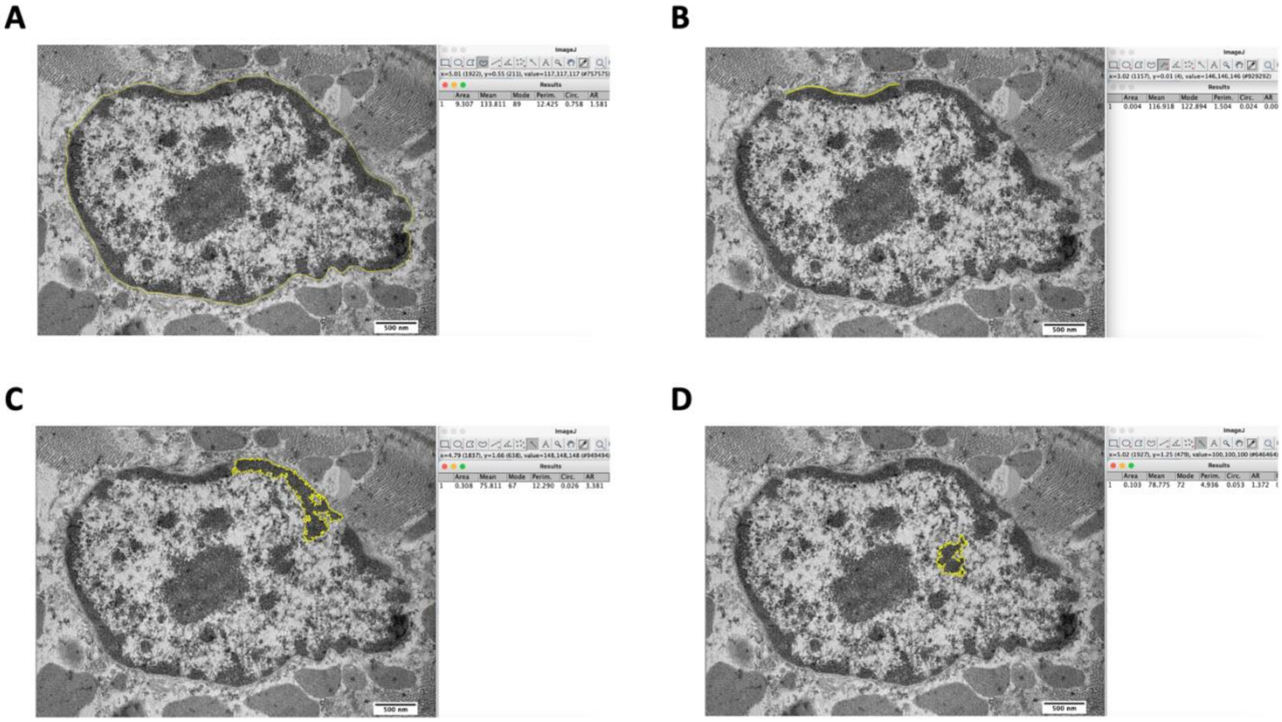

Figure S2
